# Exploring Nutritional Quality and Bioactive Compounds in Oat Mediterranean Landraces and Cultivars

**DOI:** 10.3390/antiox15030341

**Published:** 2026-03-09

**Authors:** Elena Prats, María Jesús Cañuelo, Carmen Tejero-Arroyo, Besma Sghaier-Hammami, Sofiene B. M. Hammami, Gracia Montilla-Bascon

**Affiliations:** 1 Spanish National Research Council (CSIC), Institute for Sustainable Agriculture (ISA), E-14004 Córdoba, Spain; elena.prats@ias.csic.es (E.P.); mjcanuelo@ias.csic.es (M.J.C.); 2 Department of Biochemistry and Molecular Biology, Campus Rabanales, University of Cordoba, E-14071 Cordoba, Spain; b12tearc@uco.es; 3 Laboratory of Bioggressors and Integrated Pest Management in Agriculture (LR14AGR02), National Agronomic Institute of Tunisia, University of Carthage, Cité Mahrajène, Tunis 1082, Tunisia; besma.sghaier@inat.ucar.tn; 4 Horticultural Sciences Laboratory (LR13AGR01), National Agronomic Institute of Tunisia, University of Carthage, Cité Mahrajène, Tunis 1082, Tunisia; sofiene.hammami@inat.ucar.tn

**Keywords:** oat, biocompounds, nutritional quality, avenanthramides, β-glucans

## Abstract

Oat (*Avena sativa* L.) is increasingly recognized as a functional food due to its unique profile of antioxidant and health-promoting compounds. Beyond its traditional role, our study reveals that Mediterranean landraces and related species harbour exceptional variability in both their nutritional and bioactive traits, offering untapped potential for functional food development. We analysed 126 genotypes, including landraces and cultivars, from 18 Mediterranean and European countries, quantifying the β-glucans, arabinoxylans, phenolic acids (soluble and cell wall bound), avenanthramides (AVAs: A, B, and C), carbon (C), nitrogen (N), C:N ratio, and protein content. The protein levels ranged from 9.5% to 18.5%, with several genotypes exceeding 17%, far above typical oat averages. The β-glucans reached clinically relevant thresholds (>5%) in multiple accessions, while the arabinoxylans surpassed 2% in selected genotypes. The phenolic acids and avenanthramides showed striking diversity, with some landraces accumulating more than 2000 µg/g of total AVAs. The species and phenology strongly influenced the grain composition. Thus, A. strigosa exhibited the highest β-glucan and avenanthramide contents, while early-heading genotypes had doubled avenanthramide levels compared to late-heading ones. A correlation analysis revealed synergistic patterns among the β-glucans, avenanthramides, and proteins, suggesting multi-component interactions that could enhance antioxidant functionality. These findings underscore the strategic value of Mediterranean oat germplasm for breeding programs targeting high-protein, fibre-rich, and antioxidant-enhanced cultivars. By exploiting this diversity, oats could play a pivotal role in preventing chronic diseases and advancing sustainable, health-oriented food systems.

## 1. Introduction

Oats, in particular *Avena sativa* L. and *Avena byzantina* K. Koch, sometimes known as the white and red oat, respectively, are the main cultivated oats. Alongside these hexaploid species, *Avena strigosa* Schreb., commonly known as the black oat, deserves attention for its exceptional rusticity and agronomic versatility. Historically cultivated across temperate regions, they have also been recognized as a cornerstone of Mediterranean agriculture, particularly over the last decades when their adaptability to marginal soils and resilience under low-input systems have become increasingly relevant [1,2]. Under harsh agroclimatic conditions oats can outperform other cereals, offering a sustainable solution for food and feed production [3]. This dual role as a staple and a functional food ingredient underscores their importance in modern diets and health-oriented markets. Globally, interest in oats has increased due to their agronomic versatility and their unique composition of bioactive compounds that confer health benefits [4].

The nutritional importance of oats lies in their balanced composition of macronutrients and micronutrients. Compared to other cereals, oat proteins constitute a higher proportion of the grain, delivering essential amino acids and influencing the carbon-to-nitrogen (C/N) ratio critical for metabolic pathways [5]. This ratio, often overlooked, plays a pivotal role in determining grain quality and physiological responses under stress conditions [6], and can be particularly relevant in Mediterranean environments where nitrogen availability can be limiting. Alongside proteins, oats contain significant levels of lipids rich in unsaturated fatty acids, vitamins, and minerals, which contribute to their superior nutritional profile [7,8].

Beyond their primary nutrients, oats are a reservoir of bioactive compounds. Oats contain the highest level of soluble non-starch polysaccharides among major cereals [9]. β-glucans, the most studied oat polysaccharides, are recognized for their cholesterol-lowering effects and glycaemic modulation [10]. These physiological effects are mainly associated with their ability to increase viscosity in the gastrointestinal tract, slowing nutrient absorption. β-glucans have also been associated with additional physiological actions, leading to improved immunity [11,12,13], complementing their role as key dietary fibre components. Arabinoxylans, another class of non-starch polysaccharides, also contribute significantly to the fibre content of oats. The regular intake of AX-rich foods has been linked to several physiological benefits, including reductions in circulating LDL cholesterol, improved colonic health, and enhanced glycaemic regulation [14,15]. Their physiological effects have been attributed to slower intestinal transit, reduced starch accessibility and lower glucose diffusion [16,17].

Phenolic compounds, including soluble and insoluble fractions, are central to the antioxidant potential of oats [18]. These molecules help mitigate oxidative stress, which has been implicated in chronic diseases such as cardiovascular disorders, diabetes, and cancer [8]. Among the soluble phenolics, avenanthramides (AVA-A, AVA-B, are AVA-C) are unique to oats and have attracted attention for their anti-inflammatory and cardioprotective properties [19,20].

The recent research has emphasized the synergistic interactions between polyphenols, in particular cell wall-bounded phenols and proteins, which form complexes that enhance stability and antioxidant capacity [21]. Studies combining β-glucans with polyphenols have also demonstrated synergistic effects [22]. Such combinations can enhance the antioxidant potential and improve the functional value of oat grain.

Mediterranean oat landraces represent an invaluable genetic resource for breeding programs aimed at improving both agronomic performance and nutraceutical quality. Unlike modern cultivars, which often prioritize yield over resilience, landraces harbour high allelic diversity conferring phenotypic variability [23,24]. Their genomic structure, often associated with geographical regions, reflects historical selection pressures and local adaptation, offering opportunities for the targeted introgression of desirable traits [25]. However, further efforts are needed to identify the genotypes with high nutraceutical value.

In this context, our work aims to characterize the nutritional and bioactive compound profiles of a comprehensive collection of Mediterranean oat landraces and cultivars to identify sources of high protein and health-promoting compounds, particularly β-glucans, arabinoxylans, phenolics, and avenanthramides, for application in the food industry and plant breeding initiatives. This approach aligns with global efforts to enhance crop quality, promote sustainable agriculture, and address the growing demand for functional foods that support human health.

## 2. Materials and Methods

### 2.1. Plant Material

A total of 126 accessions, including white and red oats (*A. sativa* and *A. byzantina*, respectively) and *A. strigosa*, were selected from a larger panel of 712 Mediterranean landraces and European cultivars previously characterized at agronomic, phenotypic, and genetic levels [1]. This subset was chosen to maximize the genetic diversity and to represent the 3 previously identified clusters [1], as is described in Supplementary Table S1.

All the accessions were purified through two generations of single-seed descent prior to the genotypic and phenotypic evaluations. The seeds were obtained from the Centro de Recursos Fitogenéticos (INIA, Madrid, Spain) and the United States Department of Agriculture (Washington, DC, USA). The cultivars were provided by various institutions [1,2] and by Semillas Batlle S.A. (Barcelona, Spain). These cultivars were included for comparative purposes, as they are widely grown in the Mediterranean region.

### 2.2. Sample Preparation

The plants were cultivated in Santaella (Cordoba, Spain, 37°32′45.8″ N, 4°52′14.2″ W) during the 2016/2017 growing season following an alpha-lattice design. The grains were harvested and stored at 4 °C in darkness until further use. Prior to the chemical analyses, a representative sub-sample from each lot was dehulled and ground using a Retsch mm400 (Restch, Düsseldorf, Germany) equipped with 25 mL stainless-steel vials and stainless-steel grinding ball (RETSCH). The samples were milled for 30 s at maximum speed to obtain a homogeneous particle size and then passed through a 0.5 mm (or 0.25 mm) screen. The whole-grain flour was stored at −20 °C until analysis.

### 2.3. Chemical Analysis

#### 2.3.1. Elemental Analysis of Carbon and Nitrogen

The carbon (C) and nitrogen (N) contents in the oat flour samples were determined using an elemental analyser (EuroVector EA3000, EuroVector S.A., Milan, Italy) equipped with Callidus software (version 5.1). The instrument was calibrated with acetanilide as the reference material following standard procedures for a combustion-based elemental analysis.

Prior to analysis, the samples were oven-dried, and approximately 0.2 mg of each was weighed into a tin (Sn) capsule. Combustion took place under an oxygen-enriched atmosphere, and the released gases were quantified by thermal conductivity detection at 90 °C. The results were expressed as the percentages of C and N calculated on a dry-weight basis. Three independent biological replicates per accession were analysed.

The carbon-to-nitrogen (C:N) ratio was calculated directly from the elemental composition data. The protein content was estimated from the nitrogen concentration using a nitrogen-to-protein conversion factor of 5.83, as recommended for oat grains by the FAO and by Mariotti et al. [26,27]. This cereal-specific factor provides a more accurate estimation of protein content in oat kernels [26].

#### 2.3.2. β-Glucans

The β-glucan content was quantified using a Mixed-Linkage β-Glucan Assay Kit (Megazyme, Bray, Ireland), following the microplate adaptation described by Newell et al. [28] with minor modifications. Briefly, 50 mg of flour was dispersed in 50 μL of 50% aqueous ethanol (*v*/*v*) and extracted in 1 mL of 20 mM sodium phosphate buffer (pH 6.5) using brief heating cycles (1 min) at 100 °C. After equilibration at 50 °C for 5 min, 50 μL of lichenase (10 U) was added, and the samples were vortexed and incubated for 1 h at 50 °C with mixing every 10–15 min. The reaction was stopped with 1250 μL of 200 mM sodium acetate buffer (pH 4.0) and the mixture was equilibrated at room temperature for 5 min before centrifugation at 5000× *g* for 10 min. Aliquots of 10 μL of the supernatant were incubated with 10 μL of β-glucosidase (0.02 U) in 10 mM sodium acetate buffer (pH 4.0). The plates were incubated at 50 °C for 10 min, followed by the addition of 250 µL of GOPOD reagent and further incubated for an additional 20 min. The absorbance was measured at 510 nm using a BIOTEK Synergy HT spectrophotometer (Agilent, Santa Clara, CA, USA) within 1 h. The β-glucan content (% *w*/*w*) was calculated according to the kit instructions. Three independent biological and two technical replicates per accession were analysed.

#### 2.3.3. Arabinoxylan

The arabinoxylan content was determined using a D-Xylose (Xylan & Arabinoxylan) Assay Kit (Megazyme, Bray, Ireland), a colorimetric procedure widely applied for quantifying arabinoxylans in cereal flours [29]. Approximately 100 mg of oat flour was hydrolysed with 3 mL of 1.3 M HCl at 80 °C for 10 min, vortexed, cooled for 5 min at room temperature, and neutralized with 3 mL of 1.3 M NaOH. After phase separation, 10 µL of the supernatant was transferred to a 96-well microplate, and processed according to the kit’s instructions. The absorbance was measured at 340 nm using a BIOTEK Synergy HT spectrophotometer (Agilent, Santa Clara, CA, USA). The results were expressed as g/100 g of grain flour. Three independent biological and two technical replicates per accession were analysed.

#### 2.3.4. Phenolic Compounds

The free phenolic compounds were extracted by mixing 50 mg of oat flour with 0.5 mL of methanol in 1.5 mL microcentrifuge tubes and incubating the suspension in a thermomixer (Optic Ivyme systems, Biotech, Barcelona, Spain) at 395× *g* for 20 min at room temperature. After centrifugation at 20,100× *g* for 10 min at 20 °C, the supernatant was collected. The extraction was repeated, and the supernatants were pooled for subsequent analysis.

The wall-bound phenolics were extracted from the remaining pellet by alkaline hydrolysis. Briefly, 0.4 mL of 2 M NaOH was added, and the samples were mixed and incubated at 50 °C for 1.5 h, with intermittent vortexing. After cooling, the mixture was acidified with 0.4 mL of concentrated HCl (37% *w*/*w*) and centrifuged at 39,400× *g* for 20 min. The supernatant was transferred to clean tubes.

Both the free and bound phenolic contents were determined using the Folin–Ciocalteu method [30] adapted to a 96-well microplate format [31]. The absorbance was measured at 760 nm using a microplate BIOTEK Synergy HT spectrophotometer (Agilent, Santa Clara, CA, USA). The quantification was performed using external calibration curves prepared with gallic acid (Sigma-Aldrich, Buchs SG, Switzerland). The results were expressed as mg gallic acid equivalents (GAE) per g of whole-grain flour. Three independent biological and two technical replicates per accession were analysed. This method provides an overall estimation of total phenolic content, although it does not distinguish the individual phenolic compounds. Given the unique relevance of avenanthramides in oats, these phenolic compounds were additionally quantified by HPLC.

#### 2.3.5. Avenanthramides

Analytical-grade methanol and ethanol were purchased from Sigma-Aldrich (Buchs SG, Switzerland). HPLC-grade acetonitrile was obtained from PanReac AppliChem (Barcelona, Spain). Formic acid (≥99%) was sourced from Scharlab S.L (Barcelona, Spain). Avenanthramide standards (AVA-A, AVA-B, and AVA-C; purity >98%) were obtained from ResearChem GmbH (Burgdorf, Switzerland) and verapamil hydrochloride (internal standard) from Sigma-Aldrich (Buchs SG, Switzerland). Milli-Q water was used for all aqueous solutions.

The extraction conditions were based on previously published protocols [32,33], with minor modifications. Briefly, 0.5 g of oat flour was extracted with 1.5 mL of 80% aqueous methanol (*v*/*v*). Five 2 mm glass beads and 100 µL of internal standard solution were added to each sample. The tubes were vortexed, sonicated for 10 min, and incubated in a water bath at 50 °C for 1.5 h, with intermittent vortexing. The extracts were centrifuged at 9800× *g* for 10 min, transferred to 1.5 mL microcentrifuge tubes, filtered through 0.25 µm membranes (Labbox (Branchia), Düsseldorf, Germany), and stored at −20 °C until the chromatographic analysis.

The avenanthramides were quantified using a modified HPLC method based on Pridal et al. (2018) [34]. The analyses were performed on an Agilent 2100 Series HPLC system equipped with an InfinityLab Poroshell 120 SB-C18 column (4.6 × 100 mm; 2.7 µm) and matching guard column, maintained at 38 °C. Mobile phase A consisted of water with 0.1% (*v*/*v*) formic acid and 0.05% (*v*/*v*) acetonitrile; mobile phase B was acetonitrile with 0.1% (*v*/*v*) formic acid. The gradient program was: 0 min, 20% B; 5 min, 25% B; 10 min, 25% B; 15 min, 65% B; 19 min, 100% B; 20 min, 25% B; and 25 min, 20% B. The flow rate was set at 0.6 mL/min, and 20 µL of sample was injected manually. The detection wavelengths were set to 340 nm for the avenanthramides and 280 nm for the verapamil. The quantification was performed using external calibration curves. The results were expressed as µg/g of dry weight. Three independent biological replicates were analysed per genotype.

#### 2.3.6. Non-Enzymatic Antioxidant Activity

The non-enzymatic antioxidant capacity of oat grains was assessed using methanolic extracts prepared from 20 mg of dried, finely ground material. The samples were extracted with 0.4 mL of methanol for 30 min and subsequently centrifuged at 20,100× *g* for 10 min. The free radical scavenging activity was quantified using a 2,2-diphenyl-1-picrylhydrazyl (DPPH) assay following the procedure originally described by Blois (1958), adapted to 96-well microplates [35]. Briefly, 30 µL of methanolic extract was mixed with 150 µL of 0.1 mM DPPH solution. The reaction mixture was incubated at room temperature for 30 min in the dark. The decrease in absorbance associated with the colour transition from purple to yellow was measured spectrophotometrically at 517 nm. The DPPH radical scavenging activity was calculated according to the following equation:

DPPH scavenging activity (%) = (1 − Abscontrol Abssample) × 100

#### 2.3.7. Statistical Analysis

For ease of understanding, the raw percentage mean is presented in the figures when it corresponds. However, the percentage data were normalized using an arcsine square root transformation to stabilize the variances, with the transformed value = 180/π × arcsin (√(%/100)), expressed in degrees. The data were subjected to analysis of variance (ANOVA using R software) [36], and the residual plots were inspected to confirm that the data conformed to normality. Differences between the means were assessed using Scheffé’s contrast analysis.

In addition to the ANOVA, Pearson correlation coefficients were calculated to evaluate the relationships among the grain bioactive compounds. Correlation matrices were visualized using color-coded heatmaps. Hierarchical clustering was performed to group the genotypes based on similarities in their compositional and bioactive profiles. Clustering was conducted in R using the pheatmap package (Version 1.0.13), applying the Euclidean distance as the dissimilarity metric and Ward’s minimum variance method (ward.D2) for agglomeration. Heatmaps were generated to visualize the cluster structure and trait accumulation patterns across genotypes. Boxplots and additional comparative analyses were also generated in R to illustrate the variability across genotypes.

## 3. Results

### 3.1. Distribution Patterns of Nutritional and Functional Compounds Across Oat Accessions

**Figure 1:**
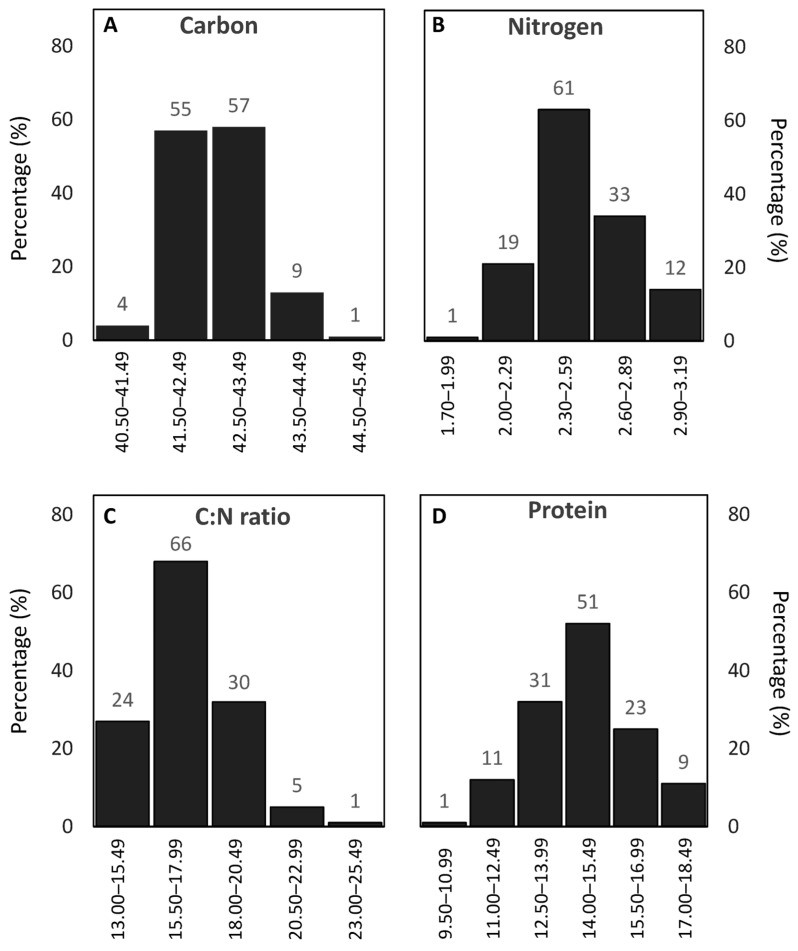
Frequency distribution of carbon content, protein concentration, and C/N ratio among Mediterranean oat genotypes. (**A**) Carbon content (%) intervals: 40.50–41.49, 41.50–42.49, 42.50–43.49, 43.50–44.49, 44.50–45.49; (**B**) Nitrogen content (%) intervals: 1.70–1.99, 2.00–2.29, 2.30–2.59, 2.60–2.89, 2.90–3.19; (**C**) C/N ratio distribution intervals: 13.00–15.49, 15.50–17.99, 18.00–20.49, 20.50–22.99, 23.00–25.49; (**D**) Protein concentration: 9.50–10.99, 11.00–12.49, 12.50–13.99, 14.00–15.49, 15.50–16.99, 17.00–18.49. The numbers above the bars indicate the number of genotypes within each interval for the corresponding trait.

The carbon content among the evaluated oat genotypes ranged from 40.5% to 45.5%, with most genotypes clustering between 41.5% and 43.5% (Figure 1A). The nitrogen (Figure 1B) and protein (Figure 1D) concentrations exhibited a broader variation, with the protein content ranging from 9.5% to 18.5%, and a modal class between 14.0% and 15.5%. Notably, 11 genotypes exceeded 17% protein, highlighting their nutritional value and a substantial phenotypic variability in this trait (Figure 1D). The C/N ratio varied from 13 to 25, with the majority of genotypes falling between 15.5 and 17.9, skewing the distribution left and suggesting that nitrogen contributed more strongly to this ratio than carbon (Figure 1C).

**Figure 2:**
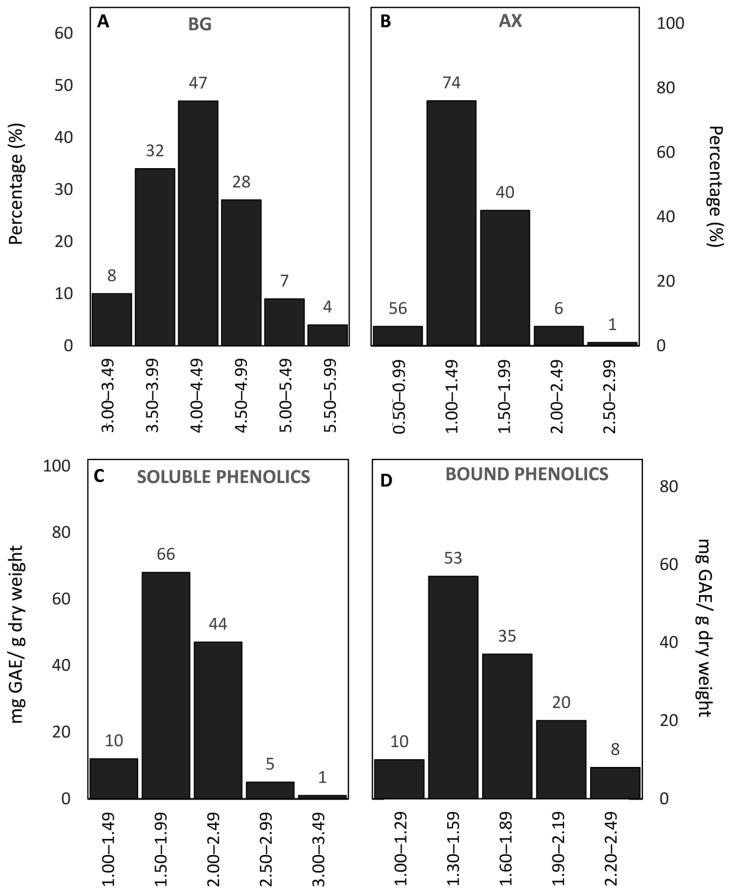
Frequency distribution of non-starch polysaccharides (β-glucans and arabinoxylans) and total phenolics (soluble and cell wall bound) among Mediterranean oat genotypes. (**A**) Β-glucans (BGs) (%) intervals: 3.00–3.49, 3.50–3.99, 4.00–4.49, 4.50–4.99, 5.00–5.49, 5.50–5.99. (**B**) Arabinoxylans (AXs) (%) intervals: 0.50–0.99, 1.00–1.49, 1.50–1.99, 2.00–2.49, 2.50–2.99. (**C**) Soluble phenolics (mg GAE/g) intervals: 1.00–1.49, 1.50–1.99, 2.00–2.49, 2.50–2.99, 3.00–3.49. (**D**) Cell wall-bound phenolics (mg GAE/g) intervals: 1.00–1.29, 1.30–1.59, 1.60–1.89, 1.90–2.19, 2.20–2.49. The numbers above the bars indicate the number of genotypes within each interval for the corresponding trait.

The content of non-starch polysaccharides, in particular β-glucans and arabinoxylans, which contribute to the dietary fibre fraction, followed a slightly left-skewed normal distribution. The β-glucan content ranged between 3 and 6%, with only four of the more than 130 genotypes in the range corresponding to a higher content. Despite this, around 30% of the genotypes had higher values than the modal (Figure 2A). The arabinoxylan content was lower than that of β-glucan, ranging from 0.5% to 2.9%, with the majority of genotypes between 1.0% and 1.9%, and seven genotypes surpassing 2.0% (Figure 2B). The phenolic compounds, which play a key role in antioxidant capacity and stress tolerance, exhibited considerable variability among the studied genotypes (Figure 2C,D). The soluble phenolics ranged from 1.0 to 3.5 mg GAE/g, showing a broader distribution compared to the cell wall-bound phenolics, which varied between 1.0 and 2.5 mg GAE/g. Notably, more than 20% of genotypes displayed cell wall-bound phenolic contents above 2.0 mg GAE/g, indicating substantial diversity in the antioxidant-related traits within these genotypes.

**Figure 3:**
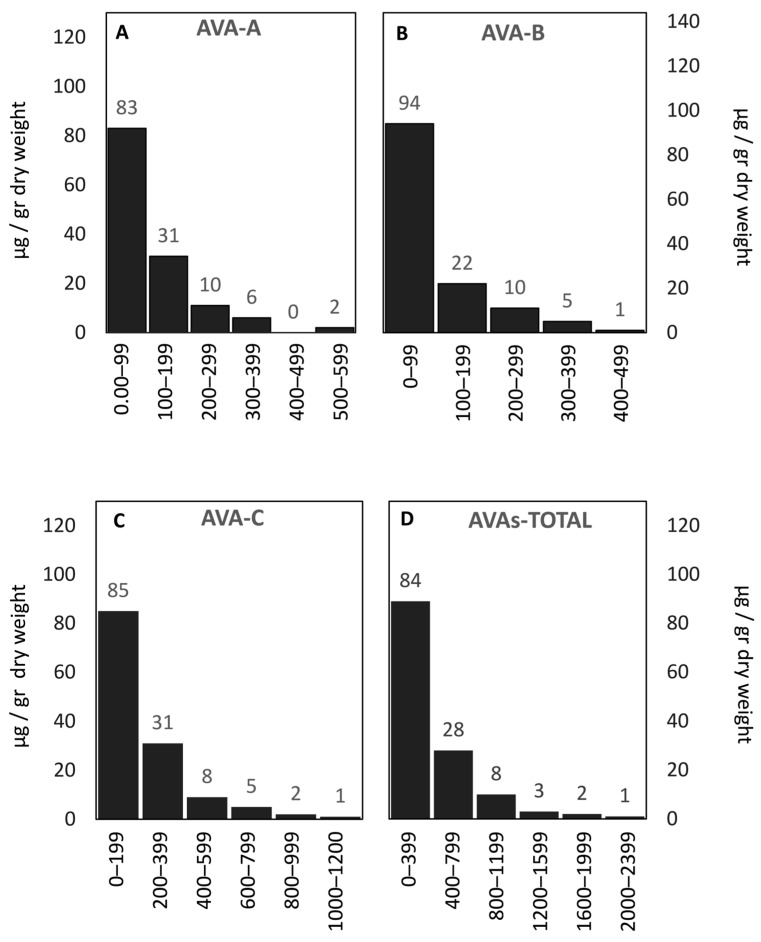
Frequency distribution of avenanthramides (A, B, C and total) among Mediterranean oat genotypes (µg/g dry weight). (**A**) Avenanthramide A (AVA-A) intervals: 0–99, 100–199, 200–299, 300–399, 400–499, 500–599; (**B**) Avenanthramide B (AVA-B) intervals: 0–99, 100–199, 200–299, 300–399, 400–499; (**C**) Avenanthramide C (AVA-C) intervals: 0–199, 200–399, 400–599, 600–799, 800–999, 1000–1200; (**D**) Total avenanthramides (AVAs-TOTAL) intervals: 0–399, 400–799, 800–1199, 1200–1599, 1600–1999, 2000–2399. The numbers above the bars indicate the number of genotypes within each interval for the corresponding trait.

The distribution of avenanthramide content among the Mediterranean oat genotypes showed a consistent pattern across the three major forms (AVA-A, AVA-B, and AVA-C) and their total concentrations (Figure 3A–D). All avenanthramides exhibited strongly left-skewed frequency distributions, indicating that most genotypes accumulated low-to-moderate levels of these compounds in the grain. AVA-A and AVA-B were predominantly below 200 µg/g dry weight, with only a few genotypes exceeding 400 µg/g. AVA-C displayed a broader range, reaching up to 1200 µg/g, nearly twice the maximum observed for AVA-A and AVA-B. Overall, between 10% and 15% of genotypes accumulated levels above the modal class for each avenanthramide, highlighting the presence of high-accumulating genotypes within the population.

### 3.2. Variation in Grain Nutritional and Functional Compounds Across Heading Date, Species, Material Type and Origin

**Figure 4:**
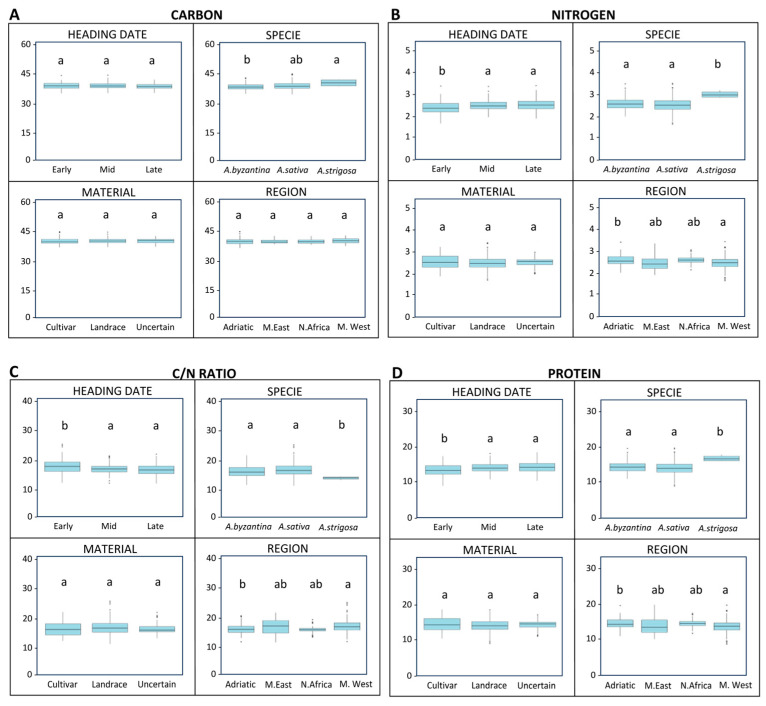
Boxplots showing variations in carbon, nitrogen, C/N ratio, and protein content among Mediterranean oat genotypes according to heading date, plant material, species, and region of origin. (**A**) Carbon content (%), (**B**) Nitrogen content (%), (**C**) C/N ratio, and (**D**) Protein concentration (%). Boxes represent interquartile ranges, horizontal lines indicate medians, and whiskers show minimum and maximum values. Letters above boxes denote statistical groupings: groups that share at least one letter do not differ significantly, whereas groups with different letters are significantly different according to Tukey’s test (*p* < 0.05).

The carbon accumulation in the grain was not significantly affected by the heading date, as the early-, mid- and late-heading genotypes showed comparable carbon levels (Figure 4A). Likewise, no differences were observed between the plant material types (landraces vs. cultivars) or among regions of landrace collection, which were defined according to previous data [1]. In contrast, species differences were evident. *A. strigosa* exhibited significantly higher carbon content than *A. byzantina* (*p* < 0.001), a trend that was also observed for the nitrogen and protein concentrations, with an average increase of more than 2% in protein content (Figure 4B,D). As expected, the nitrogen and protein contents were strongly correlated, and the early-heading genotypes displayed significantly lower values for both traits (*p* < 0.001). Regional variation was also apparent, with landraces from the west Mediterranean region showing a slight (≈1%) but significant reduction in protein content compared to those from the Adriatic region. These differences in nitrogen and protein were reflected in the C/N ratio, which varied significantly across the species, heading date, and region (Figure 4C).

**Figure 5:**
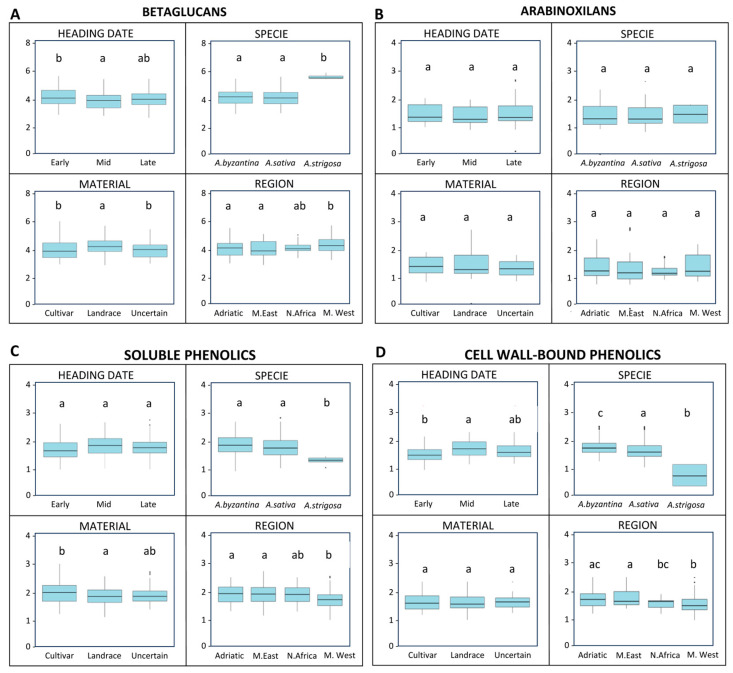
Boxplots showing variations in non-starch polysaccharides (β-glucans and arabinoxylans) and total phenolics (soluble and cell wall bound) contents among Mediterranean oat genotypes according to heading date, plant material, species, and region of origin. (**A**) Β-glucans (BGs) (%), (**B**) Arabinoxylans (AXs), (**C**) Soluble phenolics, and (**D**) Cell wall-bound phenolics. Boxes represent interquartile ranges, horizontal lines indicate medians, whiskers show minimum and maximum values. Letters above boxes denote statistical groupings: groups that share at least one letter do not differ significantly, whereas groups with different letters are significantly different according to Tukey’s test (*p* < 0.05).

The two non-starch polysaccharides analysed in this study exhibited contrasting patterns (Figure 5A,B). The arabinoxylan content remained stable across all the factors considered, heading date, species, plant material, and region, indicating a limited genetic or environmental influence on this trait. In contrast, the β-glucan concentration showed significant variation for all factors. The early-heading genotypes accumulated significantly higher β-glucan levels compared to the mid- and late-heading ones (*p* < 0.05). Species differences were pronounced, with A. strigosa displaying an increase of more than 1% in β-glucan content relative to A. sativa and A. byzantina (*p* < 0.001). The plant material also influenced the β-glucan levels, as the landraces exhibited significantly higher concentrations than the cultivars (*p* = 0.002). Regional variation was evident, with accessions from the western Mediterranean region showing the highest β-glucan content (*p* < 0.001) (Figure 5A).

The phenolic compounds (Figure 5C,D) also varied according to the factors considered, although the soluble and cell wall-bound phenolics behaved differently. The soluble phenolics were unaffected by the heading date overall, but the early-heading genotypes had significantly lower concentrations of cell wall-bound phenolics compared to the mid- and late-heading ones (*p* = 0.004). The plant material influenced the soluble phenolics, with the cultivars showing a modest but significant increase compared to the landraces (*p* = 0.005), but not the cell wall-bound phenolics. Both phenolic fractions differed by species and region. *A. strigosa* exhibited markedly reduced levels, approximately 23% lower for soluble phenolics and 38% lower for cell wall-bound phenolics (*p* < 0.001). The accessions from the western Mediterranean region also showed significantly lower phenolic contents in both fractions compared to those of other regions (*p* < 0.001).

**Figure 6:**
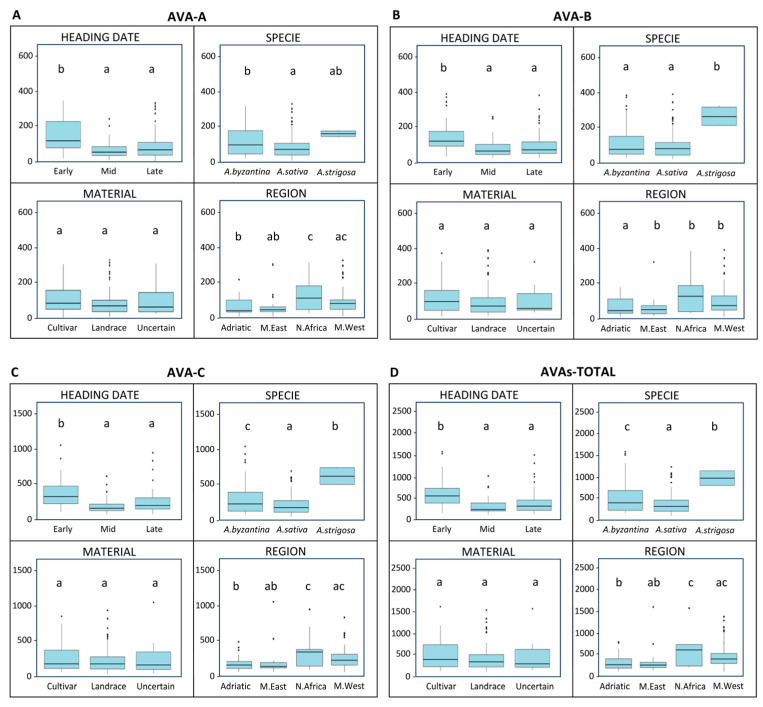
Boxplots showing variation in avenanthramides (A, B, C and Total) content among Mediterranean oat genotypes according to heading date, plant material, species, and region of origin. (**A**) Avenanthramide A (AVA-A), (**B**) Avenanthramide B (AVA-B), (**C**) Avenanthramide C (AVA-C), and (**D**) Total avenanthramides (AVAs-TOTAL). Boxes represent interquartile ranges, horizontal lines indicate medians, and whiskers show minimum and maximum values. Letters above boxes denote statistical groupings: groups that share at least one letter do not differ significantly, whereas groups with different letters are significantly different according to Tukey’s test (*p* < 0.05).

The avenanthramide content (AVA-A, AVA-B, AVA-C, and total) exhibited a consistent pattern of variation across the factors considered, except for the plant material type, where no significant differences were detected between the landraces and cultivars (Figure 6A–D). The heading date had a strong effect: the early-heading genotypes accumulated more than twice the avenanthramide content compared to the mid- and late-heading ones, which did not differ significantly from each other. Species differences were also evident, with *A. strigosa* showing the highest levels overall (*p* < 0.001). For AVA-A, the increase was moderate (27% and 84% higher than *A. byzantina* and *A. sativa*, respectively), while AVA-B and AVA-C exhibited more pronounced differences, ranging from 1.8- to 2.7-fold higher in *A. strigosa*. Significant differences between *A. sativa* and *A. byzantina* were also observed for AVA-A, AVA-C, and total avenanthramides (*p* < 0.05), with *A. byzantina* showing overall higher levels than *A. sativa*. Regional variation was also marked, with the accessions from North Africa and the western Mediterranean region showing the highest avenanthramide concentrations across all forms (*p* < 0.001).

### 3.3. Trait Correlation Patterns

**Figure 7:**
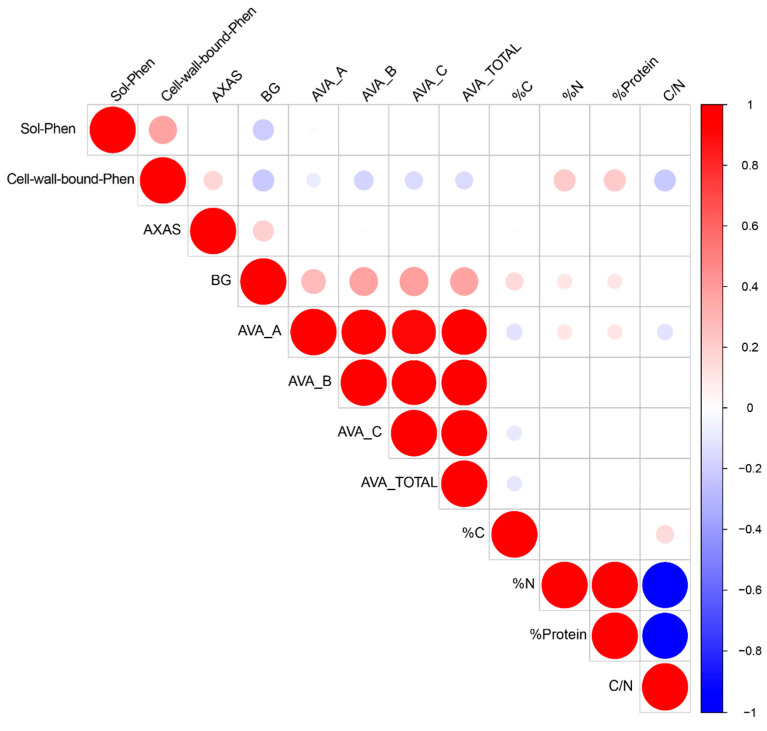
Correlation matrix showing Pearson correlation coefficients among grain composition traits and bioactive compounds in Mediterranean oat genotypes. Cells display correlation values (r), with colour intensity indicating strength and direction (blue = negative correlation, red = positive correlation). All correlations shown are significant, with at least *p* < 0.05.

The correlation analysis revealed strong and significant associations among several traits (Figure 7). As expected, the nitrogen and protein contents were perfectly correlated. Carbon and nitrogen were also significantly associated with the C/N ratio; however, the ratio was more strongly influenced by nitrogen (r = –0.97) than by carbon (r = 0.14). The phenolic compounds showed a moderate positive correlation between the soluble and cell wall-bound fractions (r = 0.36, *p* < 0.001).

The cell wall-bound phenolics exhibited broader connectivity than the soluble ones, correlating significantly with the non-starch polysaccharides, avenanthramides, and nitrogen/protein content. Specifically, they were linked to the non-starch polysaccharides in opposite directions: they were positively associated with the arabinoxylans but negatively with the β-glucans (r ≈ −0.20), and with the avenanthramides to a lesser yet significant extent. The soluble phenolics also correlated negatively with the β-glucans (r = −0.20, *p* < 0.001). The β-glucans displayed a relatively strong positive correlation with all the avenanthramides (r ≈ 0.40, *p* < 0.001), while the avenanthramides themselves were highly intercorrelated (r > 0.90, *p* < 0.001). The carbon content showed weak, albeit significant negative correlations (r ≈ −0.10) with the avenanthramides, suggesting a limited interaction between the structural carbon allocation and these bioactive compounds.

### 3.4. Clustering Patterns of Nutritional and Functional Compounds

**Figure 8:**
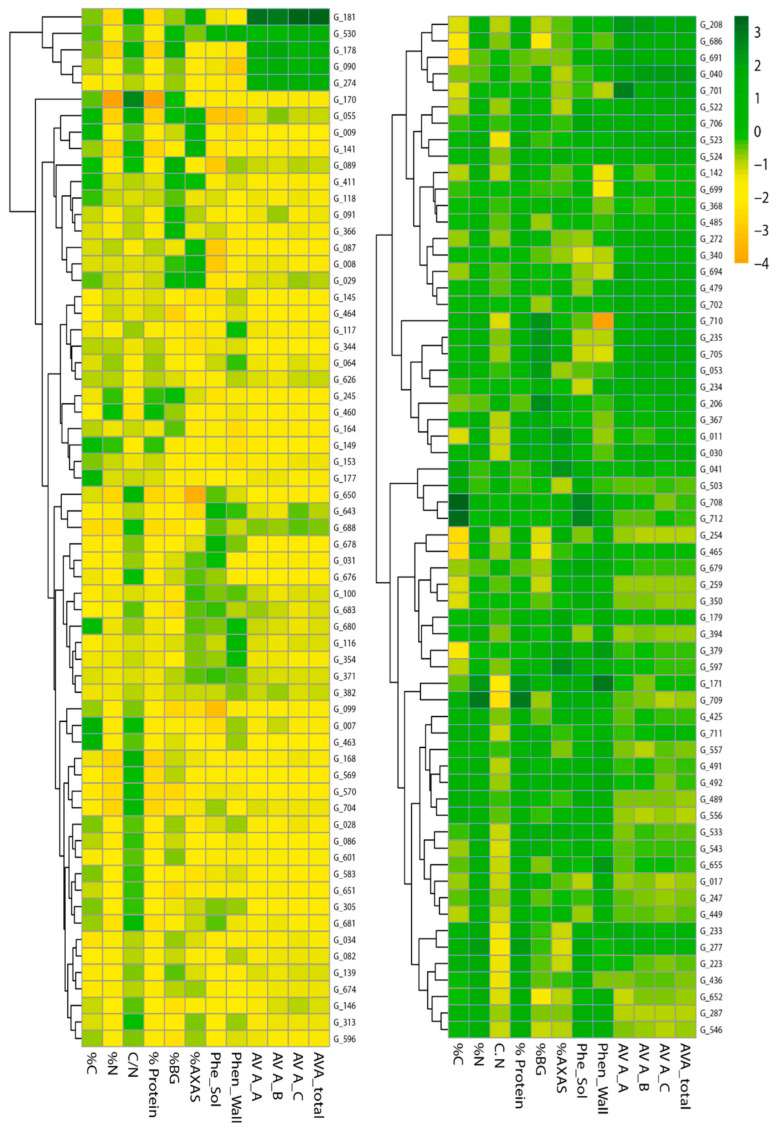
Heatmaps showing clustering of Mediterranean oat genotypes based on grain composition and bioactive compound profiles. Colour intensity represents relative concentrations of carbon, nitrogen, protein, C/N ratio, β-glucans, arabinoxylans, phenolic compounds (soluble and cell wall bound), and avenanthramides (A, B, C, and total). Colours range from orange (lowest values) through yellow and light green to dark green (highest values). Hierarchical clustering groups genotypes with similar accumulation patterns; labels indicate genotype codes.

The heatmaps in Figure 8 provide a visual overview of the clustering patterns among Mediterranean oat genotypes based on their grain composition and bioactive compound profiles. The colour gradients indicate relative concentrations, allowing for the identification of genotypes with similar accumulation patterns and those with extreme values.

The hierarchical clustering reveals two main groups of Mediterranean oat genotypes that differ in their overall accumulation patterns of nutritional and bioactive traits. One cluster comprises accessions with generally lower values across most components, whereas the opposite cluster includes genotypes enriched in several key traits. Within this structure, sub-clusters highlight more specific combinations: for instance, genotypes such as G_181, G_530, G_178, and G_090 display comparatively high avenanthramide contents relative to the other compounds, whereas the genotypes including accessions G_208, G_686, G_691, G_040, and G_701 show a broader enrichment pattern, combining elevated phenolics, avenanthramides, and protein. Another subgroup, represented by genotypes such as G_710, G_235, or G_705, exhibits concurrent increases in β-glucans, arabinoxylans, and protein. These configurations underscore the presence of biologically meaningful trait profiles within the Mediterranean germplasm, revealing genotypes with complementary nutritional and bioactive characteristics that may be valuable for different breeding objectives.

### 3.5. Antioxidant Potential

**Figure 9:**
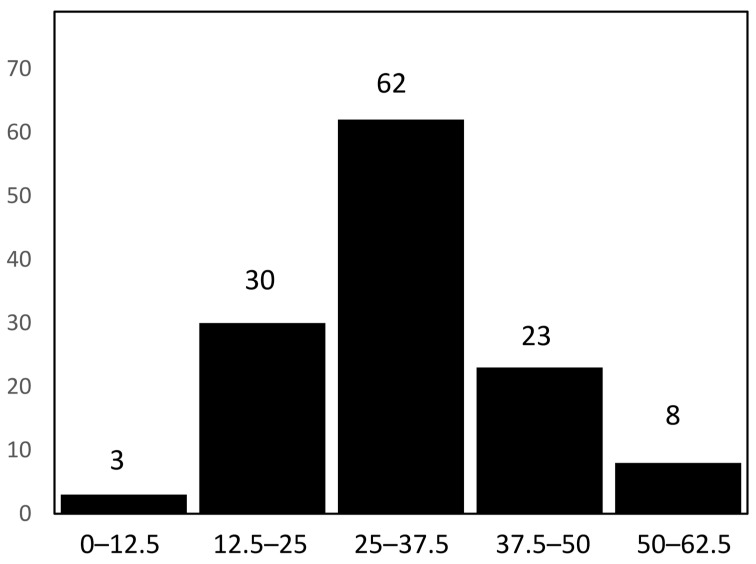
Frequency distribution of non-enzymatic antioxidant activity estimated through 2, 2-diphenyl-1-picrylhydrazyl (DPPH) free radical scavenging activity (%). Intervals: 0–12.5, 12.5–25, 25–37.5, 37.5–50, 50–62.5 Numbers above bars indicate number of genotypes within each interval.

The non-enzymatic antioxidant activity quantified through the DPPH assay exhibited substantial variability among the 126 oat genotypes evaluated (Figure 9). The DPPH radical scavenging capacity ranged from low to moderately high values, with a distribution that was clearly skewed towards the intermediate classes. Most genotypes clustered within the 12.5–37.5% inhibition range, whereas only a small proportion of around 2% showed values below 12.5%, and 15 genotypes exceeded 50% inhibition.

To explore the biochemical basis underlying this variation, Pearson correlation analyses were performed between the DPPH activity and the major classes of phenolic compounds. A strong and highly significant positive correlation was detected between the DPPH scavenging capacity and soluble phenolics (r = 0.56, *p* < 0.001), indicating that the genotypes with higher concentrations of extractable phenolics tend to display greater non-enzymatic antioxidant potential. The cell wall-bound phenolics also showed a positive association with the DPPH activity (r = 0.26, *p* = 0.002), although weaker than that observed for soluble phenolics. In contrast, no significant correlations were found between the DPPH activity and individual avenanthramides (AVA-A, AVA-B, AVA-C) or with the total AVAs, suggesting that within this germplasm set, avenanthramides contributed little to the overall variability in the DPPH radical scavenging capacity when compared with the total phenolic constituents.

## 4. Discussion

Grain quality encompasses the macronutrient composition and bioactive compounds that collectively determine the nutritional and functional value of cereals. Oats stand out among major cereals for their combination of high protein content and a distinctive profile of health-promoting compounds, such as β-glucans, arabinoxylans, phenolic acids, and avenanthramides. These compounds underpin oats’ well-documented metabolic, antioxidant and anti-inflammatory benefits [7,8,19], positioning oats as a strategic crop for functional food development, particularly in Mediterranean environments where nutritional enhancement and resilience are key priorities [11,15].

The broad variability observed in the grain protein across the Mediterranean germplasm, with a subset of genotypes clearly above the typical oat average content, reflects diverse physiological strategies for nitrogen uptake, allocation and storage [37,38]. Such diversity is highly valuable for breeding programs, as it provides accessions suited to different nutritional goals and production contexts [39]. The genotypes with the highest N levels can be leveraged to improve nutritional profile of grains destined for human consumption, whereas others maybe more suited to feed applications, where specific C/N balances are advantageous. The presence of contrasting physiological patterns within the same collection highlights the adaptability of Mediterranean oats and reinforces their potential for selection programmes aimed at quality differentiation.

Beyond macronutrients, the pronounced variation in bioactive compounds highlights the functional potential of Mediterranean oat landraces and cultivars. Several accessions exhibited β-glucan concentrations above the threshold associated with clinically relevant effects on cholesterol reduction and glycaemic control, as recognized by EFSA health claims [40]. The arabinoxylan content, although generally lower, also surpassed the reported average in a subset of genotypes, contributing to dietary fibre functionality and potential synergy with β-glucans to promote gut health [16]. The soluble phenolics slightly exceeded typical cereal values, suggesting enhanced antioxidant potential [41,42]. The avenanthramides, unique to oats, displayed a distribution consistent with previous reports [43,44,45,46], including a subset of genotypes with clearly elevated content, indicative of a more active biosynthetic pathway and relevant for developing cultivars with improved anti-inflammatory and cardioprotective properties [47].

Previous studies have shown that differences in growth cycle (early- vs. late-maturing varieties) and species can significantly affect the protein concentration, fibre components, and phenolic profiles in cereals [39,48]. In our analysis, the absence of variation in the carbon content across maturity groups and regions suggests that the carbon allocation to grains is relatively stable, consistent with previous findings in oats and other cereals [37]. Conversely, the nitrogen and protein content differed among species and maturity groups, reflecting the influence of genetic background and phenology. As expected, *A. strigosa* exhibited higher protein levels than *A. sativa* [39]. Overall, the early-heading genotypes tended to accumulate less protein, consistent with the trade-off between accelerated development and nitrogen assimilation, a pattern documented in cereals under stress-prone environments [48]. On the other hand, the early-flowering genotypes, including those of *A. strigosa*, showed higher avenanthramide and, to a lesser extent, phenolic contents. This pattern is physiologically plausible because earlier heading exposes plants to the increasing irradiance, temperature fluctuations and onset of water limitation typical of late spring in Mediterranean climates during grain filling. These conditions activate the stress-responsive metabolic pathways that stimulate the phenylpropanoid cascade, enhancing the biosynthesis of phenolics and avenanthramides via the induction of key enzymes, such as PAL, CCoAOMT and HHT [48,49]. Moreover, the shorter grain-filling period characteristic of early-heading materials may favour the allocation of metabolic resources toward defence-related secondary metabolites rather than prolonged deposition of storage reserves. The consistently elevated avenanthramide content observed in *A. strigosa*, a species with earlier phenology and strong responsiveness to environmental cues, supports the notion that phenology–stress interactions play a central role in shaping the accumulation of these compounds [50].

Regional differences could be linked to local adaptation and soil fertility, reinforcing the importance of conserving genetic diversity for breeding programs targeting nutritional improvement. The contrasting behaviour of arabinoxylans and β-glucans underscores the complexity of non-starch polysaccharide regulation in oats. The stability of arabinoxylan content across the different studied factors suggests a strong canalization of this trait, consistent with previous findings in cereals [49,50]. Conversely, the marked variability in the β-glucan concentration aligns with reports highlighting its sensitivity to genotype and growing conditions [38,39]. The high avenanthramide levels in North African and western Mediterranean landraces likely reflect adaptation to environments characterized by abiotic stress, which is known to stimulate phenolic accumulation [47]. These findings underscore the need to consider both environmental drivers and developmental timing when selecting genotypes for functional food applications.

The correlations among grain components provide further insights for breeding and processing strategies. Most of the correlations observed in this study might be explained by the spatial distribution of compounds within the grain, although the metabolic trade-offs during grain filling might also contribute [38,51]. The strong positive correlation between arabinoxylans and esterified phenolics aligns with their co-localization in the outer grain layers, where phenolic acids reinforce cell walls through cross-linking and contribute to antioxidant defence [52,53]. Conversely, β-glucans, concentrated in the endosperm and sub-aleurone, show negative correlations with the wall-bound phenolics, as these tissues are less lignified and do not require phenolic cross-links for structural integrity [22,54]. The protein content correlates positively with the cell wall-bound phenolics, consistent with their co-localization in the aleurone layer, and the formation of fibre–protein complexes, which influence digestibility, glycaemic response and antioxidant interactions during processing [55,56]. The positive correlation of avenanthramides with β-glucans and a slight negative correlation with cell wall-bound phenolics is consistent with their localization and biosynthetic regulation, since avenanthramides accumulate in inner tissues rich in β-glucans and are involved in defence responses, whereas esterified phenolics dominate in outer layers [47,57].

From a breeding perspective, these interactions are critical because improving one component may have synergistic or antagonistic effects on others due to shared developmental pathways or tissue-specific localization. Multi-trait selection strategies and genomic approaches that incorporate these correlations are therefore essential to optimize both agronomic performance and health-promoting attributes [51,58,59]. The clustering analysis revealed distinct accumulation profiles across genotypes, confirming the feasibility of identifying materials that combine desirable levels of protein, fibre, and antioxidant components, and supporting their value for the development of high-functionality oat-based products [47,50].

The non-enzymatic antioxidant capacity assessed through the DPPH assay is largely driven by the pool of readily extractable, electron-donating phenolics, which agrees with the correlation and with the co-enrichment patterns observed in the heatmap. This strong alignment with soluble phenolics is chemically expected in a methanolic single-electron/hydrogen atom transfer assay and is physiologically consistent with the spatial partitioning of phenolic compounds within oat grains. In this framework, the weak association between DPPH and avenanthramides might be also expected since, although AVAs possess recognized redox activity, they tend to track β-glucans and inner endosperm tissues rather than the broader phenolic background, a pattern that aligns with their low correlation values [54]. In addition, phenolic cross-linking to cell wall polysaccharides and the formation of phenolic–protein or β-glucan–polyphenol complexes can limit extractability while maintaining coordinated variation among genotypes, thereby reinforcing the correlations within phenolic fractions without necessarily amplifying the DPPH response [22]. Taken together, the correlation matrix and clustering patterns support a coherent interpretation of antioxidant functionality in oats, where the soluble phenolics emerge as the dominant contributors to the overall antioxidant potential, the wall-bound phenolics influence this capacity indirectly through shared phenylpropanoid regulation and tissue co-localization, and the avenanthramides exert a more context-dependent role shaped by their compartmentation and macromolecular associations.

Overall, the findings of this study not only highlight the substantial variability in nutritional and functional traits among Mediterranean oat genotypes but also emphasize the strategic value of these resources for breeding programs targeting health-oriented markets. The integration of genetic insights and nutritional data provides a robust foundation for developing oat-based products that combine functionality, sustainability, and health benefits, meeting the demands of both consumers and future food systems.

## Data Availability

The raw data supporting the conclusions of this article will be made available by the authors on request.

## Supplementary Materials

The following supporting information can be downloaded at: https://www.mdpi.com/article/10.3390/antiox15030341/s1, Table S1: Available information on the oat collection used in this study.

## Abbreviations

The following abbreviations are used in this manuscript:

AVN	Avenanthramide
AXs	Arabinoxylans
C	Carbon
N	Nitrogen

## References

[1] Canales F.J., Montilla-Bascon G., Bekele W.A., Howarth C.J., Langdon T., Rispail N., Tinker N.A., Prats E. (2021). Population genomics of Mediterranean oat (*A. sativa*) reveals high genetic diversity and three loci for heading date. Theor. Appl. Genet..

[2] Sanchez-Martin J., Rubiales D., Flores F., Emeran A.A., Shtaya M.J.Y., Sillero J.C., Allagui M.B., Prats E. (2014). Adaptation of oat (*Avena sativa*) cultivars to autumn sowings in Mediterranean environments. Field Crops Res..

[3] Stevens E.J., Armstrong K.W., Bezar H.J., Griffin W.B., Suttie J.M., Reynolds S.G. (2004). Fodder oats: An overview. Fodder Oats: A World Overview.

[4] Zhang K., Dong R., Hu X., Ren C., Li Y. (2021). Oat-based foods: Chemical constituents, glycemic index, and the effect of processing. Foods.

[5] Zheng Z.L. (2009). Carbon and nitrogen nutrient balance signaling in plants. Plant Signal. Behav..

[6] Xie J., Liu M., Xiao Z., Li X., Cao F., Chen J., Huang M., Ali I., Iqbal A., Wahab A. (2025). Relationships between grain quality and leaf carbon and nitrogen status in high-quality hybrid rice across different sowing dates and nitrogen management. Front. Agron..

[7] Paudel D., Dhungana B., Caffe M., Krishnan P. (2021). A review of health-beneficial properties of oats. Foods.

[8] Tang Y., Li S., Yan J., Peng Y., Weng W., Yao X., Gao A., Cheng J., Ruan J., Xu B. (2023). Bioactive components and health functions of oat. Food Rev. Int..

[9] Bai J., Li T., Zhang W., Fan M., Qian H., Li Y., Wang L. (2021). Systematic assessment of oat β-glucan catabolism during in vitro digestion and fermentation. Food Chem..

[10] Zhang M., Liang Y., Pei Y., Gao W., Zhang Z. (2009). Effect of process on physicochemical properties of oat bran soluble dietary fiber. J. Food Sci..

[11] Singh R.P., Bhardwaj A. (2023). β-glucans: A potential source for maintaining gut microbiota and the immune system. Front. Nutr..

[12] Vetvicka V., Dvorak B., Vetvickova J., Richter J., Krizan J., Sima P., Yvin J.C. (2007). Orally administered marine (1→3)-β-D-glucan Phycarine stimulates both humoral and cellular immunity. Int. J. Biol. Macromol..

[13] Zekovic D.B., Kwiatkowski S., Vrvic M.M., Jakovljevic D., Moran C.A. (2005). Natural and modified (1→3)-β-D-glucans in health promotion and disease alleviation. Crit. Rev. Biotechnol..

[14] Kellow N.J., Walker K.Z. (2018). Authorised EU health claim for arabinoxylan. Foods, Nutrients and Food Ingredients with Authorised EU Health Claims.

[15] Rosicka-Kaczmarek J., Komisarczyk A., Nebesny E., Makowski B. (2016). The influence of arabinoxylans on the quality of grain industry products. Eur. Food Res. Technol..

[16] Chen Z., Li S., Fu Y., Li C., Chen D., Chen H. (2019). Arabinoxylan structural characteristics, interaction with gut microbiota and potential health functions. J. Funct. Foods.

[17] EFSA Panel on Dietetic Products, Nutrition and Allergies (2011). Scientific opinion on the substantiation of health claims related to arabinoxylan produced from wheat endosperm and reduction of post-prandial glycaemic responses (ID 830) pursuant to Article 13(1) of Regulation (EC) No 1924/2006. EFSA J..

[18] Ryan L., Thondre P.S., Henry C.J.K. (2011). Oat-based breakfast cereals are a rich source of polyphenols and high in antioxidant potential. J. Food Compos. Anal..

[19] Xie X., Lin M.Y., Xiao G.S., Liu H.F., Wang F., Liu D.J., Ma L.K., Wang Q., Li Z.Y. (2024). Phenolic amides (avenanthramides) in oats—An update review. Bioengineered.

[20] Kang C., Shin W.S., Yeo D., Lim W., Zhang T., Ji L.L. (2018). Anti-inflammatory effect of avenanthramides via NF-κB pathways in C2c12 skeletal muscle cells. Free Radic. Biol. Med..

[21] Muntaha S.T., Rakha A., Rasheed H., Fatima I., Butt M.S., Abdi G., Aadil R.M. (2025). Polyphenol-protein particles: A nutraceutical breakthrough in nutrition and food science. J. Agric. Food Res..

[22] Wu Z., Ming J., Gao R., Wang Y., Liang Q., Yu H., Zhao G. (2011). Characterization and antioxidant activity of the complex of tea polyphenols and oat β-glucan. J. Agric. Food Chem..

[23] Canales F.J., Montilla-Bascon G., Gallego-Sanchez L.M., Flores F., Rispail N., Prats E. (2021). Deciphering main climate and edaphic components driving oat adaptation to Mediterranean environments. Front. Plant Sci..

[24] Montilla-Bascon G., Sanchez-Martin J., Rispail N., Rubiales D., Mur L., Langdon T., Griffiths I., Howarth C., Prats E. (2013). Genetic diversity and population structure among oat cultivars and landraces. Plant Mol. Biol. Report..

[25] Newton A.C., Akar T., Baresel J.P., Bebeli P.J., Bettencourt E., Bladenopoulos K.V., Czembor J.H., Fasoula D.A., Katsiotis A., Koutis K. (2010). Cereal landraces for sustainable agriculture. A review. Agron. Sustain. Dev..

[26] FAO (2002). Protein Conversion Factors.

[27] Mariotti F., Tome D., Mirand P.P. (2008). Converting nitrogen into protein—Beyond 6.25 and Jones’ factors. Crit. Rev. Food Sci. Nutr..

[28] Newell M.A., Kim H.J., Asoro F.G., Lauter A.M., White P.J., Scott M.P., Jannink J.L. (2014). Microenzymatic evaluation of oat (*Avena sativa* L.) β-glucan for high-throughput phenotyping. Cereal Chem..

[29] Balakrishnan A.P., Jain A., Singh S.K., Ahlawat A.K., Jaiswal J.P., Mahendru-Singh A., Bhardwaj R. (2025). Development of a NIRS-based prediction model for measurement of whole wheat flour arabinoxylan content to aid rapid germplasm screening. J. Cereal Sci..

[30] Singleton V.L. (1985). Citation classic-colorimetry of total phenolics with phosphomolybdic-phosphotungstic acid reagents. Curr. Contents/Agric. Biol. Environ. Sci..

[31] Bobo-Garcia G., Davidov-Pardo G., Arroqui C., Virseda P., Marin-Arroyo M.R., Navarro M. (2015). Intra-laboratory validation of microplate methods for total phenolic content and antioxidant activity on polyphenolic extracts, and comparison with conventional spectrophotometric methods. J. Sci. Food Agric..

[32] Bryngelsson S., Mannerstedt-Fogelfors B., Kamal-Eldin A., Andersson R., Dimberg L.H. (2002). Lipids and antioxidants in groats and hulls of Swedish oats (*Avena sativa* L). J. Sci. Food Agric..

[33] Wise M.L. (2011). Effect of chemical systemic acquired resistance elicitors on avenanthramide biosynthesis in oat (*Avena sativa*). J. Agric. Food Chem..

[34] Pridal A.A., Böttger W., Ross A.B. (2018). Analysis of avenanthramides in oat products and estimation of avenanthramide intake in humans. Food Chem..

[35] Blois M.S. (1958). Antioxidant determinations by the use of a stable free radical. Nature.

[36] R Development Core Team (2008). R: A Language and Environment for Statistical Computing.

[37] Doehlert D.C., McMullen M.S., Hammond J.J. (2001). Genotypic and environmental effects on grain yield and quality of oat grown in North Dakota. Crop Sci..

[38] Peterson D.M., Wesenberg D.M., Burrup D.E., Erickson C.A. (2005). Relationships among agronomic traits and grain composition in oat genotypes grown in different environments. Crop Sci..

[39] Howarth C.J., Martinez-Martin P.M.J., Cowan A.A., Griffiths I.M., Sanderson R., Lister S.J., Langdon T., Clarke S., Fradgley N., Marshall A.H. (2021). Genotype and environment affect the grain quality and yield of winter oats (*Avena sativa* L.). Foods.

[40] EFSA Panel on Dietetic Products, Nutrition and Allergies (2010). Scientific opinion on dietary reference values for carbohydrates and dietary fibre. EFSA J..

[41] Dykes L., Rooney L.W. (2007). Phenolic compounds in cereal grains and their health benefits. Cereal Foods World.

[42] Horvat D., Simic G., Drezner G., Lalic A., Ledencan T., Tucak M., Plavsic H., Andric L., Zdunic Z. (2020). Phenolic acid profiles and antioxidant activity of major cereal crops. Antioxidants.

[43] Leonova S., Gnutikov A., Loskutov I., Blinova E., Gustafsson K.E., Olsson O. (2020). Diversity of avenanthramide content in wild and cultivated oats. Proc. Appl. Bot. Genet. Breed..

[44] Lee J.K., Kim I., Jeon E.K., Ha J.H., Hwang C.W., Kim J.C., Yang W.S., Choi H., Kim H.D., Kim C.H. (2022). Bacterially converted oat active ingredients enhances antioxidative and anti-uvb photoaging activities. Evid.-Based Complement. Altern. Med..

[45] Peterson D.M., Hahn M.J., Emmons C.L. (2002). Oat avenanthramides exhibit antioxidant activities in vitro. Food Chem..

[46] Redaelli R., Dimberg L., Germeier C.U., Berardo N., Locatelli S., Guerrini L. (2016). Variability of tocopherols, tocotrienols and avenanthramides contents in European oat germplasm. Euphytica.

[47] Meydani M., YiFang C. (2013). Avenanthramides, unique polyphenols of oats with potential health effects. Oats Nutrition and Technology.

[48] Peltonen-Sainio P., Kangas A., Salo Y., Jauhiainen L. (2007). Grain number dominates grain weight in temperate cereal yield determination: Evidence based on 30 years of multi-location trials. Field Crops Res..

[49] Izydorczyk M.S., Biliaderis C.G. (1995). Cereal arabinoxylans: Advances in structure and physicochemical properties. Carbohydr. Polym..

[50] Wood P.J. (2010). Oat and rye β-glucan: Properties and function. Cereal Chem..

[51] Dwivedi S.L., Reynolds M.P., Ortiz R. (2021). Mitigating tradeoffs in plant breeding. Iscience.

[52] Mnich E., Bjarnholt N., Eudes A., Harholt J., Holland C., Jorgensen B., Larsen F.H., Liu M., Manat R., Meyer A.S. (2020). Phenolic cross-links: Building and de-constructing the plant cell wall. Nat. Prod. Rep..

[53] Schendel R.R., Meyer M.R., Bunzel M. (2016). Quantitative profiling of feruloylated arabinoxylan side-chains from graminaceous cell walls. Front. Plant Sci..

[54] Peterson D.M. (2001). Oat antioxidants. J. Cereal Sci..

[55] Calinoiu L.F., Vodnar D.C. (2018). Whole grains and phenolic acids: A review on bioactivity, functionality, health benefits and bioavailability. Nutrients.

[56] Guan H., Zhang W.Y., Sun-Waterhouse D., Jiang Y., Li F., Waterhouse G.N., Li D.P. (2021). Phenolic-protein interactions in foods and post ingestion: Switches empowering health outcomes. Trends Food Sci. Technol..

[57] Dimberg L.H., Sunnerheim K., Sundberg B., Walsh K. (2001). Stability of oat avenanthramides. Cereal Chem..

[58] Bazzer S.K., Oliveira G., Fiedler J.D., Nandety R.S., Jannink J.L., Caffe M. (2025). Genomic strategies to facilitate breeding for increased β-Glucan content in oat (*Avena sativa* L.). BMC Genom..

[59] Dhakal A., Poland J., Adhikari L., Faryna E., Fiedler J., Rutkoski J.E., Arbelaez J.D. (2024). Implementing multi-trait genomic selection to improve grain milling quality in oats (*Avena sativa* L.). Plant Genome.

