# Supplementary material for: Exploring Nutritional Quality and Bioactive Compounds in Oat Mediterranean Landraces and Cultivars
Source: Antioxidants (Basel). 2026 Mar 9;15(3):341. doi: 10.3390/antiox15030341 (PMC13024331; doi:10.3390/antiox15030341)
Supplement: Supplementary file 1 [file antioxidants-15-00341-s001.zip › antioxidants-4130574-supplementary.pdf]

Supplementary material

Exploring Nutritional Quality and Bioactive Compounds in Oat Mediterranean Landraces and Cultivars

Elena Prats [1], María Jesús Cañuelo [1], Carmen Tejero-Arroyo [2], Besma Sghaier-Hammami [3], Sofiene B. M. Hammami [4] and Gracia Montilla-Bascon [1,*]

[1] Spanish National Research Council (CSIC), Institute for Sustainable Agriculture (ISA), E-14004 Córdoba, Spain; elena.prats@ias.csic.es (E.P.); mjcanuelo@ias.csic.es (M.J.C.)
[2] Department of Biochemistry and Molecular Biology, Campus Rabanales, University of Cordoba, E-14071 Cordoba, Spain; b12tearc@uco.es
[3] Laboratory of Bioggressors and Integrated Pest Management in Agriculture (LR14AGR02), National Agronomic Institute of Tunisia, University of Carthage, Cité Mahrajène, Tunis 1082, Tunisia; besma.sghaier@inat.ucar.tn
[4] Horticultural Sciences Laboratory (LR13AGR01), National Agronomic Institute of Tunisia, University of Carthage, Cité Mahrajène, Tunis 1082, Tunisia; sofiene.hammami@inat.ucar.tn

* Correspondence: gmontilla@ias.csic.es

*to whom correspondence should be addressed:

Gracia Montilla-Bascon

CSIC, Institute for Sustainable Agriculture,

Avda. Menéndez Pidal, s/n, Córdoba - 14004, Spain.

gmontilla@ias.csic.es

Supplementary Table S1. Available information on the oat collection used in this study.

GENOTYPE	Bank Code	Other name	Specie	Material	Region of collection	Heading date
**G_006**	BGE008119	--	*A.sativa*	Landrace	Spain	Early
**G_007**	BGE008121	--	*A.sativa*	Landrace	Spain	Mid
**G_008**	BGE008122	--	*A.sativa*	Landrace	Spain	Late
**G_009**	BGE008123	--	*A.sativa*	Landrace	Spain	Early
**G_011**	BGE008140	--	*A.byzantina*	Landrace	Spain	Mid
**G_017**	BGE008179	--	*A.sativa*	Landrace	Spain	Mid
**G_028**	BGE008400	--	*A.byzantina*	Landrace	Spain	Mid
**G_029**	BGE008401	--	*A.sativa*	Landrace	Spain	Early
**G_030**	BGE008402	--	*A.sativa*	Landrace	Spain	Mid
**G_031**	BGE008403	--	*A.sativa*	Landrace	Spain	Mid
**G_034**	BGE008406	--	*A.sativa*	Landrace	Spain	Early
**G_040**	BGE008479	--	*A.sativa*	Landrace	Spain	Late
**G_041**	BGE008480	--	*A.sativa*	Landrace	Spain	Mid
**G_053**	BGE009644	--	*A.sativa*	Landrace	Spain	Mid
**G_055**	BGE009646	--	*A.sativa*	Landrace	Spain	Early
**G_064**	BGE009746	--	*A.sativa*	Landrace	Spain	Late
**G_082**	BGE010432	--	*A.sativa*	Landrace	Spain	Late
**G_086**	BGE010499	--	*A.sativa*	Landrace	Spain	Early
**G_087**	BGE010500	--	*A.sativa*	Landrace	Spain	Mid
**G_089**	BGE010502	--	*A.sativa*	Landrace	Spain	Mid
**G_090**	BGE010503	--	*A.sativa*	Landrace	Spain	Early
**G_091**	BGE010504	--	*A.sativa*	Landrace	Spain	Early
**G_099**	BGE015359	--	*A.byzantina*	Landrace	Spain	Early
**G_100**	BGE015360	--	*A.byzantina*	Landrace	Spain	Mid
**G_116**	BGE018492	--	*A.sativa*	Landrace	Spain	Mid
**G_117**	BGE018495	--	*A.sativa*	Landrace	Spain	Mid
**G_118**	BGE018498	--	*A.sativa*	Landrace	Spain	Mid
**G_139**	BGE026964	--	*A.sativa*	Landrace	Spain	Mid
**G_141**	BGE030934	--	*A.sativa*	Landrace	Spain	Mid
**G_142**	BGE005494	--	*A.byzantina*	Landrace	Spain	Mid
**G_145**	BGE008131	--	*A.sativa*	Landrace	Spain	Mid
**G_146**	BGE008142	--	*A.sativa*	Landrace	Spain	Mid
**G_149**	BGE008625	--	*A.sativa*	Landrace	Spain	Mid
**G_153**	BGE008629	--	*A.sativa*	Landrace	Spain	Late
**G_164**	BGE008640	--	*A.sativa*	Landrace	Spain	Late
**G_168**	BGE008644	--	*A.sativa*	Landrace	Spain	Early
**G_170**	BGE008646	--	*A.sativa*	Landrace	Spain	Early
**G_171**	BGE008647	--	*A.byzantina*	Landrace	Spain	Mid

**G_177**	BGE008653	--	*A.sativa*	Landrace	Spain	Late
**G_178**	BGE008654	--	*A.sativa*	Landrace	Spain	Early
**G_179**	BGE008655	--	*A.sativa*	Landrace	Spain	Early
**G_181**	BGE008657	--	*A.sativa*	Landrace	Spain	Mid
**G_206**	BGE009767	--	*A.byzantina*	Landrace	Spain	Early
**G_208**	BGE009774	--	*A.byzantina*	Landrace	Spain	Mid
**G_223**	BGE018466	--	*A.sativa*	Landrace	Spain	Late
**G_233**	BGE018572	--	*A.byzantina*	Landrace	Spain	Early
**G_234**	BGE018573	--	*A.byzantina*	Landrace	Spain	Late
**G_235**	BGE018574	--	*A.byzantina*	Landrace	Spain	Mid
**G_245**	BGE042532	--	*A.byzantina*	Landrace	Spain	Mid
**G_247**	Clav 1822	*Gray. CI 1822*	*A.byzantina*	Uncertain	Spain	Mid
**G_254**	PI 287338	*AVE 132/59*	*A.sativa*	Landrace	Adriatic rim	Late
**G_259**	Clav 1832	*CI 1832*	*A.byzantina*	Uncertain	North Africa	Mid
**G_272**	PI 258558	*WIR 4741*	*A.byzantina*	Landrace	North Africa	Mid
**G_274**	PI 258582	*WIR 4764*	*A.byzantina*	Landrace	North Africa	Mid
**G_277**	PI 264211	*Avoine Noire_912*	*A.sativa*	Uncertain	North Africa	Mid
**G_287**	PI 374397	*45/71-57*	*A.sativa*	Landrace	Adriatic rim	Late
**G_305**	PI 251577	*M-311*	*A.sativa*	Uncertain	Adriatic rim	Mid
**G_313**	PI 266274	*Ozimi Oves_289. WIR 10089*	*A.sativa*	Uncertain	Adriatic rim	Late
**G_340**	PI 259867	*10. Z-24*	*A.sativa*	Uncertain	Adriatic rim	Late
**G_344**	PI 259871	*14. Z-64*	*A.sativa*	Uncertain	Adriatic rim	Late
**G_350**	PI 264860	*981*	*A.sativa*	Landrace	Adriatic rim	Mid
**G_354**	PI 287396	*Z-24. AVE 662/60*	*A.sativa*	Cultivar	Adriatic rim	Late
**G_366**	PI 365622	--	*A.sativa*	Landrace	North Africa	Mid
**G_367**	PI 249721	*16991*	*A.sativa*	Uncertain	Adriatic rim	Mid
**G_368**	PI 249932	*F46832*	*A.sativa*	Uncertain	Adriatic rim	Mid
**G_371**	Clav 9100	*CW 542. CI 9100*	*A.byzantina*	Landrace	Adriatic rim	Mid
**G_379**	PI 258579	*WIR 10202*	*A.byzantina*	Landrace	Adriatic rim	Mid
**G_382**	PI 258585	*WIR 4793*	*A.byzantina*	Landrace	Adriatic rim	Mid
**G_394**	PI 264846	*440*	*A.sativa*	Landrace	Adriatic rim	Mid
**G_411**	PI 287273	*AVE 145/62*	*A.byzantina*	Landrace	Adriatic rim	Late
**G_425**	PI 287292	*AVE 499/62*	*A.byzantina*	Landrace	Adriatic rim	Mid
**G_436**	PI 287351	*AVE 265/59*	*A.sativa*	Landrace	Adriatic rim	Mid
**G_449**	Clav 8090	*CI 8090*	*A.sativa*	Cultivar	Middle East	Mid
**G_460**	PI 110260	*Soldanelle Primaverile. 232. Clav 3276*	*A.sativa*	Uncertain	Adriatic rim	Mid
**G_463**	PI 564725	*Lidia*	*A.sativa*	Cultivar	Adriatic rim	Early
**G_464**	Clav 2864	*Black_Belgium. CI 2864*	*A.sativa*	Cultivar	Adriatic rim	Mid
**G_465**	Clav 2866	*White Oat of Scotland*	*A.sativa*	Uncertain	Adriatic rim	Mid

**G_479**	PI 287295	*AVE 539/62*	*A.byzantina*	Landrace	Adriatic rim	Mid
**G_485**	PI 264207	*Palestine*	*A.sativa*	Uncertain	North Africa	Early
**G_489**	PI 344830	*36*	*A.sativa*	Landrace	Adriatic rim	Late
**G_491**	PI 344833	*39*	*A.sativa*	Landrace	Adriatic rim	Mid
**G_492**	PI 344834	*40*	*A.sativa*	Landrace	Adriatic rim	Late
**G_503**	PI 362367	*28/B*	*A.sativa*	Landrace	Adriatic rim	Late
**G_522**	PI 55524	*Red Algerian. Rousse. Clav 2017*	*A.byzantina*	Landrace	North Africa	Mid
**G_523**	PI 158213	*WIR 1732. Clav_4783*	*A.byzantina*	Landrace	North Africa	Early
**G_524**	PI 189766	*Avoine Creme*	*A.sativa*	Uncertain	North Africa	Mid
**G_530**	Clav 9023	*CD 3994. CI 9023*	*A.byzantina*	Uncertain	Middle East	Early
**G_533**	PI 258567	*WIR 4864*	*A.byzantina*	Landrace	Middle East	Mid
**G_543**	Clav 1073	*Joanette. CI_1073*	*A.sativa*	Cultivar	Cultivar	Late
**G_546**	Clav 1606	*Golden Giant. CI 1606*	*A.sativa*	Cultivar	Cultivar	Late
**G_556**	PI 119476	*2018. CI 3439. Clav_3439*	*A.sativa*	Landrace	Middle East	Mid
**G_557**	PI 119477	*2101. CI 3440. Clav 3440*	*A.sativa*	Landrace	Middle East	Mid
**G_569**	PI 168078	*2161. Clav 5238. CI 5238*	*A.byzantina*	Landrace	Middle East	Mid
**G_570**	PI 168079	*2163. Clav 5239. CI 5239*	*A.byzantina*	Landrace	Middle East	Mid
**G_583**	PI 168099	*3452. Clav 5259. CI 5259*	*A.byzantina*	Landrace	Middle East	Late
**G_596**	PI 168113	*4027. Clav 5273. CI 5273*	*A.sativa*	Landrace	Middle East	Late
**G_597**	PI 168114	*4035. Clav 5274. CI 5274*	*A.sativa*	Landrace	Middle East	Mid
**G_601**	PI 168119	*4251. Clav 5279. CI 5279*	*A.sativa*	Landrace	Middle East	Late
**G_626**	PI 177840	*5356*	*A.sativa*	Landrace	Middle East	Mid
**G_643**	Clav 9101	*CW 544. CI 9101*	*A.byzantina*	Landrace	Middle East	Early
**G_650**	PI 258575	*WIR 5398*	*A.byzantina*	Landrace	Middle East	Mid
**G_651**	PI 266972	*P. No. 59/639. Kutuk 9985/1*	*A.byzantina*	Uncertain	Middle East	Mid
**G_652**	PI 340989	*Bozkir. Y-1*	*A.sativa*	Cultivar	Middle East	Mid
**G_655**	PI 341014	*Y-42*	*A.sativa*	Landrace	Middle East	Mid
**G_674**	--	*Alcudia*	*A.sativa*	Cultivar	Cultivar	Early
**G_676**	--	*Araceli*	*A.sativa*	Cultivar	Cultivar	Mid
**G_678**	--	*Caleche*	*A.sativa*	Cultivar	Cultivar	Late
**G_679**	--	*Canelle*	*A.sativa*	Cultivar	Cultivar	Mid
**G_680**	--	*Cassandra*	*A.byzantina*	Cultivar	Cultivar	Early
**G_681**	--	*Chambord*	*A.sativa*	Cultivar	Cultivar	Mid
**G_683**	--	*Charming*	*A.sativa*	Cultivar	Cultivar	Late
**G_686**	--	*Cory*	*A.sativa*	Cultivar	Cultivar	Mid

**G_688**	--	*Flega*	*A.sativa*	Cultivar	Cultivar	Early
**G_691**	--	*Hamel*	*A.sativa*	Cultivar	Cultivar	Early
**G_694**	--	*Karmela*	*A.sativa*	Cultivar	Cultivar	Early
**G_699**	--	*Orblanche*	*A.sativa*	Cultivar	Cultivar	Mid
**G_701**	--	*Patones*	*A.sativa*	Cultivar	Cultivar	Early
**G_702**	--	*Prevision*	*A.byzantina*	Cultivar	Cultivar	Early
**G_704**	--	*Rapidena*	*A.sativa*	Cultivar	Cultivar	Early
**G_705**	--	*Saia*	*A.sativa*	Cultivar	Cultivar	Mid
**G_706**	--	*Selma*	*A.sativa*	Cultivar	Cultivar	Late
**G_708**	--	*Kbira*	*A.sativa*	Cultivar	Cultivar	NA
**G_709**	--	*Forridena*	*A.sativa*	Cultivar	Cultivar	NA
**G_710**	--	*Stricta*	*A.strigosa*	Cultivar	Cultivar	NA
**G_711**	--	*Panache*	*A.strigosa*	Cultivar	Cultivar	NA
**G_712**	--	*M-77*	*A.sativa*	Cultivar	Cultivar	NA
